# The Cytotoxic Effect of Curcumin in Rhabdomyosarcoma Is Associated with the Modulation of AMPK, AKT/mTOR, STAT, and p53 Signaling

**DOI:** 10.3390/nu15030740

**Published:** 2023-02-01

**Authors:** Sara Salucci, Alberto Bavelloni, Anna Bartoletti Stella, Francesco Fabbri, Ivan Vannini, Manuela Piazzi, Karyna Volkava, Katia Scotlandi, Giovanni Martinelli, Irene Faenza, William Blalock

**Affiliations:** 1 Dipartimento di Scienze Biomediche e Neuromotorie (DIBINEM), Università di Bologna, 40126 Bologna, Italy; 2 Laboratorio di Oncologia Sperimentale, IRCCS, Istituto Ortopedico Rizzoli, 40136 Bologna, Italy; 3 Dipartimento di Medicina Specialistica, Diagnostica e Sperimentale (DIMES), Università di Bologna, 40126 Bologna, Italy; 4 Laboratorio di Bioscienze, IRCCS Istituto Romagnolo per lo Studio dei Tumori (IRST) “Dino Amadori”, 47014 Meldola, Italy; 5 ‘‘Luigi Luca Cavalli-Sforza’’ Istituto di Genetica Molecolare, Consiglio Nazionale delle Ricerca (IGM-CNR), 40136 Bologna, Italy; 6 IRCCS, Istituto Ortopedico Rizzoli, 40136 Bologna, Italy; 7 Dipartimento di Farmacia e Biotecnologie (FABIT), Università di Bologna, 40126 Bologna, Italy

**Keywords:** cancer therapy, signal transduction, drug design and targeting, nutraceuticals, muscle tissue, sarcoma

## Abstract

Approximately 7% of cancers arising in children and 1% of those arising in adults are soft tissue sarcomas (STS). Of these malignancies, rhabdomyosarcoma (RMS) is the most common. RMS survival rates using current therapeutic protocols have remained largely unchanged in the past decade. Thus, it is imperative that the main molecular drivers in RMS tumorigenesis are defined so that more precise, effective, and less toxic therapies can be designed. Curcumin, a common herbal supplement derived from plants of the *Curcuma longa* species, has an exceptionally low dietary biotoxicity profile and has demonstrated anti-tumorigenic benefits in vitro. In this study, the anti-tumorigenic activity of curcumin was assessed in rhabdomyosarcoma cell lines and used to identify the major pathways responsible for curcumin’s anti-tumorigenic effects. Curcumin treatment resulted in cell cycle arrest, inhibited cell migration and colony forming potential, and induced apoptotic cell death. Proteome profiler array analysis demonstrated that curcumin treatment primarily influenced flux through the AKT-mammalian target of rapamycin (mTOR), signal transducer and activator of transcription (STAT), AMP-dependent kinase (AMPK), and p53 associated pathways in a rhabdomyosarcoma subtype-specific manner. Thus, the strategic, combinational therapeutic targeting of these pathways may present the best option to treat this group of tumors.

## 1. Introduction

Soft tissue sarcomas (STS) comprise around 7% of cancers arising in children and 1% of those arising in adults. The most common of these STS is rhabdomyosarcoma (RMS), which comprises 50% of the STS diagnosed in children and adolescents, and is characterized by distinctive traits of skeletal muscle lineage [1]. The global disease burden of RMS varies with geographic location, having an incidence of 4.5–5.0 individuals per million in Western cultures (US and Europe) to around 2.0 individuals per million in various Asian cultures (Japan, China, India). Moreover, the odds of developing RMS were found to be significantly lowered if both parents were Hispanic, suggesting potential genetic/ethnic, environmental, and cultural/behavioral aspects to disease occurrence [1].

Rhabdomyosarcoma is classified into five histotypes. The two most common, alveolar rhabdomyosarcoma (ARMS) and embryonal rhabdomyosarcoma (ERMS), account for about 20–30% and 70–80% of all RMS, respectively [2,3,4]. ERMS is more common in early childhood with a second peak of incidence in early adolescence, while the incidence of ARMS remains constant from childhood through early adulthood. Pediatric cases of RMS are more common in males than females with a ratio of 1.5:1, while pleomorphic RMS, which occurs in mid-/late-adulthood, primarily targets males [1]. Diverse risk factors have been described for RMS development, but whether and how these factors truly contribute to disease is still inconclusive. While patients with germline syndromes such as Li-Fraumeni and Noonan syndromes develop RMS more frequently than their normal peers, only about 5% of RMS patients present with a germline syndrome co-morbidity. Additionally, studies examining the role of environmental factors in the origins of RMS are very limited, but have suggested associations between RMS and pre-natal X-ray exposure, parental recreational drug use, childhood allergies, and the use of fertility medications, among others [1].

Molecular classification divides RMS into two major subsets, fusion-positive (those harboring either fused Paired Box 3-Forkhead box O1 (PAX3-FOXO1) or the PAX7-FOXO1 transcription factors) and fusion-negative RMS (those lacking one of these signature fusions) [4,5,6]. At the molecular level, those RMS histologically identified as ARMS are typically PAX3-FOXO1 (60%) or PAX7-FOXO1 (20%) fusion-positive and are characterized by the poorest prognosis; however, promising results to arrest tumor progression have been obtained using inhibitors targeting the destabilization of the PAX3-FOXO1 oncoprotein in animal models. In contrast, fusion-negative RMS are characterized by a variety of alterations, including losses or mutations in the mouse double minute-2 homolog (MDM2)-p53 pathway, the rat sarcoma virus (RAS) pathway, and cell cycle genes, as well as chromosomal gains [3,4,7,8].

In both fusion-positive and -negative RMS, alterations are present in genes such as RAS, phosphatidylinositol-4,5-bisphosphate 3-kinase, catalytic subunit α (PIK3CA), fibroblast growth factor receptor (FGFR)-4, avian erythroblastosis oncogene (ERBB2/HER2) receptor tyrosine kinase and platelet-derived growth factor receptor (PDGFR), as well as insulin-like growth factor (IGF)-2 overexpression [9,10,11,12]. Alterations in these genes provoke the aberrant activation of the receptor tyrosine kinase (RTK)/RAS/phosphatidylinositol-3 kinase (PI3K) and RTK/RAS/mitogen activated kinase (MEK)/extracellular regulated kinase (ERK) axes. In fusion-positive RMS, the product of the PAX3-FOXO1 (or PAX7-FOXO1) gene suppresses phosphatase and tensin homolog (PTEN) expression, thereby promoting the activation of the PI3K/AKT/mTOR pathway [13].

The current protocol used to treat RMS involves a multifactorial approach including surgery, radiotherapy, and chemotherapy; however, the survival rates have remained largely unchanged in the past decade [14,15]. Thus, it is imperative that the molecular events that drive tumorigenesis are defined so that more effective, less toxic therapies can be evaluated. Curcumin is a common dietary spice of Eastern culture, derived from plants of the *Curcuma longa* species, which has achieved a certain fame as an herbal supplement mainly due to a number of potential health benefits, including anti-tumorigenic activity, reported from in vitro studies, and its low dietary biotoxicity profile [16,17]. Unfortunately, a number of studies have demonstrated that in its native form curcumin exhibits poor dietary bioavailability, never reaching clinically relevant concentrations [18]. Health benefits attributed to curcumin from in vitro studies are associated with the broad range of reactive groups present in the compound. Curcumin contains two O-methoxy phenolic ring moieties bound by an α,β unsaturated carbonyl bridge which can present as both di-keto or enol-keto forms configured in *cis* or *trans*. Thus, the reactive groups of curcumin consist of two methoxy groups, two phenolic hydroxyl groups, two phenyl rings, diketone, carbonyl, and enolic moieties in a flexible molecule capable of binding a number of macromolecular targets in the cell [16].

Curcumin was previously demonstrated to induce a G1/G0 cell cycle arrest and cell death through inhibition of mTOR, in a pair of rhabdomyosarcoma cell lines, with a reported IC50 of 2.5 µM after 6 days treatment [19]. In the study reported here, we chose to reanalyze the anti-tumorigenic effect of curcumin in three rhabdomyosarcoma cell lines, A204 (fusion-negative, p53-postitive, ERMS), SJCRH30 (RH30; PAX3-FOXO1A fusion-positive, heterozygous mutant p53, amplification of 12q13–15, ARMS), and RD (fusion-negative, MYC amplification, mutant NRAS (Q61H), homozygous mutant p53, ERMS), utilizing higher dosing and shorter time of treatment, with the following objectives in mind: (1) define what major signaling pathways are affected (directly or indirectly) by curcumin treatment in rhabdomyosarcoma for the purpose of designing better targeted therapies that individually or in combination specifically target the same pathways altered by curcumin; and (2) establish a clear idea of what pathways are influenced by curcumin in rhabdomyosarcoma in order to generate a signaling profile for validating the effectiveness of newer more bioactive/bioavailable forms of curcumin in rhabdomyosarcoma, and potentially other soft tissue sarcomas. [20].

## 2. Materials and Methods

### 2.1. Cell Lines and Reagents

Curcumin was purchased from Selleckchem (Munich, Germany) and dissolved in dimethylsulfoxide (DMSO) to generate a stock solution (50 mM) which was subsequently diluted with the medium before adding to the cells. The three rhabdomyosarcoma cell lines used in this study, A204, RD, and SJCRH30, were purchased from the American Type Culture Collection (ATCC; Manassas, VA, USA). Cell lines were routinely cultured in Dulbecco’s Modified Eagle Medium supplemented with 10% inactivated fetal calf serum (FBS; Life Technologies Corporation, Monza, Italy) and 2 mM L-glutamine (Sigma-Aldrich, St. Louis, MO, USA). Cells were maintained at 37 °C in a humidified 5% CO_2_ atmosphere.

### 2.2. Cell Proliferation Assay

Cells were seeded into 96-well plates at a density of 5000 cells/well, in 100 µL of cell culture medium, and incubated for 24 h to allow cell adherence. To test the effects of curcumin, growth media was exchanged, and the rhabdomyosarcoma cell lines were cultured for 24, 48, or 72 h, either in the presence of the vehicle (DMSO 0.1%) or increasing concentrations of curcumin (0.8–50 µM). Cell growth was determined using resazurin-based PrestoBlue reagent (Invitrogen, Monza, Italy), according to the manufacturer’s instructions. Briefly, the PrestoBlue solution (10 µL) was added to each well, and the plates were then incubated for an additional 2 h. The plates were then directly read on an Infinite M200 photometer (Tecan Group Ltd., Mannedorf, Switzerland) at a wavelength of 600 nm.

### 2.3. Trypan Blue Dye Exclusion Assay

The effect of curcumin on cell viability was determined using Trypan blue exclusion and manual counting of live and dead cells, utilizing a hemocytometer. Briefly, cells were washed and suspended (1.0 × 10^5^ cells/mL) in a solution of 1× PBS: 0.5 mM EDTA (Life Technologies) containing 0.2% BSA (Sigma-Aldrich). Fifty (50) µL of the cell suspension was taken and mixed with an equal volume of 0.4% Trypan blue. The solution was mixed thoroughly and allowed to stand for 5 min at room temperature. Ten (10) µL of the solution was transferred to a hemocytometer, and both viable (clear) and dead (blue) cells were counted. The number of live cells divided by the total number of counted cells (clear and blue) gave the percent viability.

### 2.4. Cell Cycle Analysis

To analyze cell cycle distribution, cells were treated with curcumin for 24 h, and then fixed in cold 70% ethanol at 4 °C for 24 h. The fixed cells were centrifuged at 1500 rpm for 5 min. The cell pellet was washed twice with ice-cold PBS and stained with 0.5 mL FxCycle™ PI/RNase Staining Solution (ThermoFisher Scientific, Monza, Italy). Cell cycle distribution was evaluated in 10,000 cells, using an Attune Nxt Acoustic Focusing Cytometer (Life Technologies) equipped with a blue laser (488 nm). Data were acquired in list mode using Attune Cytometric 2.6 software (Life Technologies).

### 2.5. Apoptosis Assay

Cells were treated with 20 µM curcumin for 24 h. Apoptosis was then evaluated by flow cytometry analysis using the Phycoerythrin Annexin V detection kit I (BD Biosciences, San Jose, CA, USA), exploiting the binding of PE-conjugated Annexin V for the detection of apoptotic and necrotic cells. Secondary staining with 7-amino-actinomycin D (7-AAD) allowed for the identification of early apoptotic cells. Cell staining was performed according to the manufacturer’s instructions. Fluorescence resulting from PE and 7-AAD was measured at 530 nm and 620 nm, respectively. Stained cells (10,000 cells/sample) were acquired on a Attune Nxt Acoustic Focusing Cytometer (Life Technologies), and data were analyzed using Attune Cytometric 2.6 software (Life Technologies).

### 2.6. Colony Formation Assay

Rhabdomyosarcoma cells treated with 10 µM or 20 µM curcumin or untreated (control) for 24 h were plated at 5 × 10^2^ cells/well in a six-well tissue culture plate. Cells were cultured at 37 °C in 5% CO_2_ for 10 days. Colonies were fixed with 100% methanol and stained with 0.05% (*w*/*v*) crystal violet:25% methanol. Stained plates were photographed with a Bio-Rad ChemiDoc XRS system (Bio-Rad Laboratories, Segrate (MI), Italy). Colony number and size were determined using ImageJ software.

### 2.7. Antibody Arrays

Cells were either treated or untreated with 20 µM curcumin for 24 h. Cells were collected and lysed in kit-supplied lysis buffer and precleared of cellular debri by centrifugation. Protein lysates were then quantitated using the Bio-Rad BSA Protein Reagent. For each antibody array, 500 µg of cellular extract was incubated with the Phospho-Kinase Array Kit membrane (Proteome Profiler; R&D Systems, Abingdon, United Kingdom), according to the manufacturer’s instructions. Following incubation with antibody and streptavidin–HRP conjugate, filter images were acquired on a Bio-Rad ChemiDoc XRS system (Bio-Rad). Densitometry values of spots were estimated by ImageJ software and expressed as arbitrary units. Multiple film exposures were used to verify the linearity of the samples analyzed and to avoid saturation of the film. In antibody arrays, the average signal of the pair of duplicate spots, representing each phosphorylated kinase protein, was calculated after the subtraction of background values (pixel density) from negative control spots, and normalization to average values from positive control spots.

### 2.8. Statistical Analysis

Data are presented as the mean ±SD for the indicated number of independently performed experiments (at least *n* = 3) and were analyzed by a two-tailed Student t-test or 2-way ANOVA. All statistical analyses were performed, and all graphs generated, using the GraphPad Prism, v.6.0 software (GraphPad Software, La Jolla, CA, USA).

## 3. Results

### 3.1. Curcumin Rapidly Kills Rhabdomyosarcoma Cells

**Figure 1:**
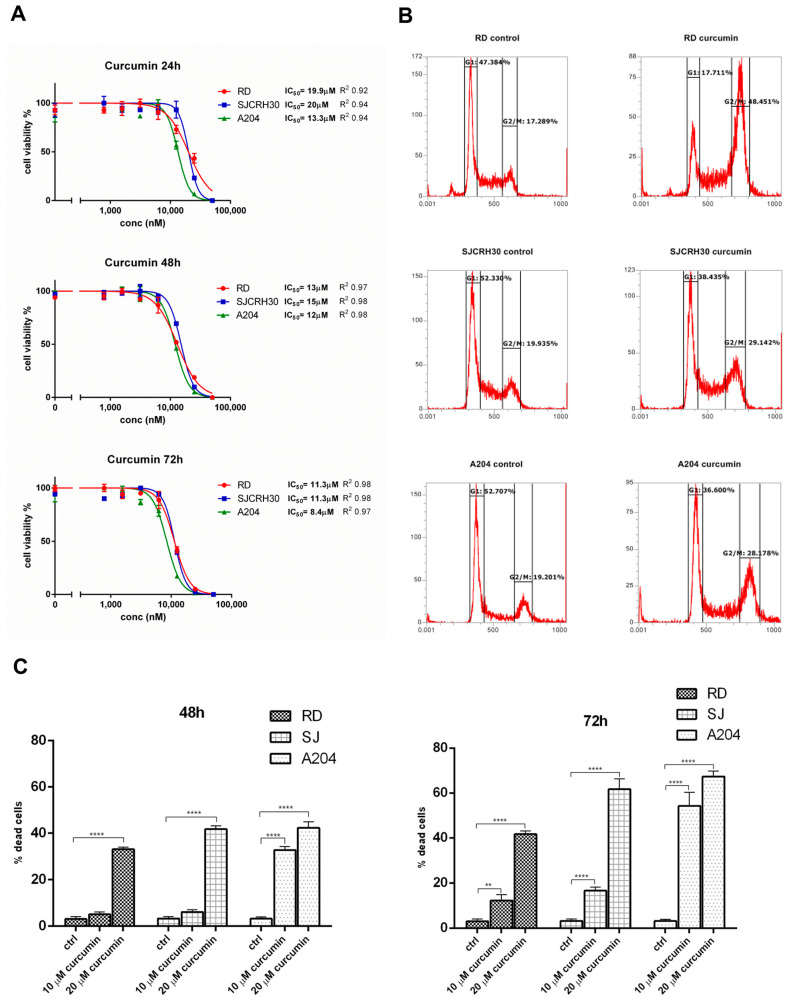
Curcumin treatment leads to decreased cell proliferation/viability and a G2/M cell cycle arrest in RMS cell lines. (**A**) The effects of curcumin on the proliferation and viability of A204, RD, and SJRH30 (RH30) cells was assayed using a resazurin-based cell proliferation/viability assay at 24, 48, and 72 h in the presence of various concentrations of curcumin (0.8–50 µM). IC50 values were obtained after 48 and 72 h treatment. Three replicates were tested per concentration and at least three independent experiments were performed (bars, ±s.d.). (**B**) Rhabdomyosarcoma cells (A204, RD, and RH30) were treated with 20 µM curcumin for 24 h and then analyzed by flow cytometry to determine the cell cycle distribution of the cells. The graph shows the percent distribution of cells in each of the cell cycle phases and is representative of three independent experiments. (**C**) The ability of curcumin to induce cell killing in RMS was measured by Trypan blue viability assay. A204, RD, and RH30 cells were treated with curcumin (10 or 20 µM) or left untreated (control) for 48 and 72 h. Both detached and attached cells were collected and stained with 0.4% Trypan blue solution and counted on a hemocytometer. Results are the mean of three different experiments ± s.d. Ctrl, Untreated cells. ** *p* < 0.01, **** *p* < 0.0001.

The antineoplastic properties of curcumin have been reported in a number of previous studies [16,17]. In rhabdomyosarcoma, curcumin was reported to block mTOR activity and to induce cell death in a p53-independent manner [19]. To examine more closely the pathways involved in curcumin mediated toxicity, the rhabdomyosarcoma cell lines, A204, SJCRH30, and RD were treated with varying dosages of curcumin for 24, 48, and 72 h, and the effects of curcumin on cell proliferation/survival were measured. Similar to previous reports, curcumin inhibited proliferation and reduced cell viability of the RMS cell lines, demonstrating an IC^50^ between 8.4 and 20 μM at 72 h. The wild-type p53 expressing ERMS cell line, A204, showed the most sensitivity, while the ERMS cell line RD and the fusion-positive ARMS cell line RH30, which both express a heterozygous and homozygous mutant p53, respectively, demonstrated less sensitivity (Figure 1A).

Cell cycle analysis was conducted to determine in what phase of the cell cycle curcumin inhibited cell proliferation. In contrast to that shown by Beevers et al. [19], cell cycle analysis demonstrated that treatment with 20 μM curcumin for 24 h resulted in a significant G2/M arrest in each of the tested cell lines with the observed G2/M arrest most noticeable in the RD cell line (RD, 17.3% vs. 48.5%; SJCRH30, 19.9% vs. 29.1%; A204, 19.2% vs. 28.2%; (control vs. treated)) (Figure 1B).

To verify whether curcumin treatment only induced arrest of cell proliferation or could also promote cell killing in the RMS cells at the tested doses, curcumin-treated RD, RH30, and A204 (48 and 72 h) were assayed for cell viability using Trypan blue. At 48 h, only the A204 cells demonstrated a significant response to the lower dose of curcumin (10 μM), while 20 μM curcumin induced a significant level of cell death in each of the tested cell lines. At 72 h, both concentrations of curcumin induced significant cell mortality in all the tested cell lines. Again, A204 demonstrated the most sensitivity to the effects of curcumin on cell survival. In all cases, the chemo-resistant RD cell line showed the most resistance to curcumin’s cell killing effects, but 20 μM curcumin still induced slightly over 35% mortality in these cells after 48 h (Figure 1C).

### 3.2. Curcumin Induces Apoptotic Cell Death in Rhabdomyosarcoma, Regardless of the Status of p53

**Figure 2:**
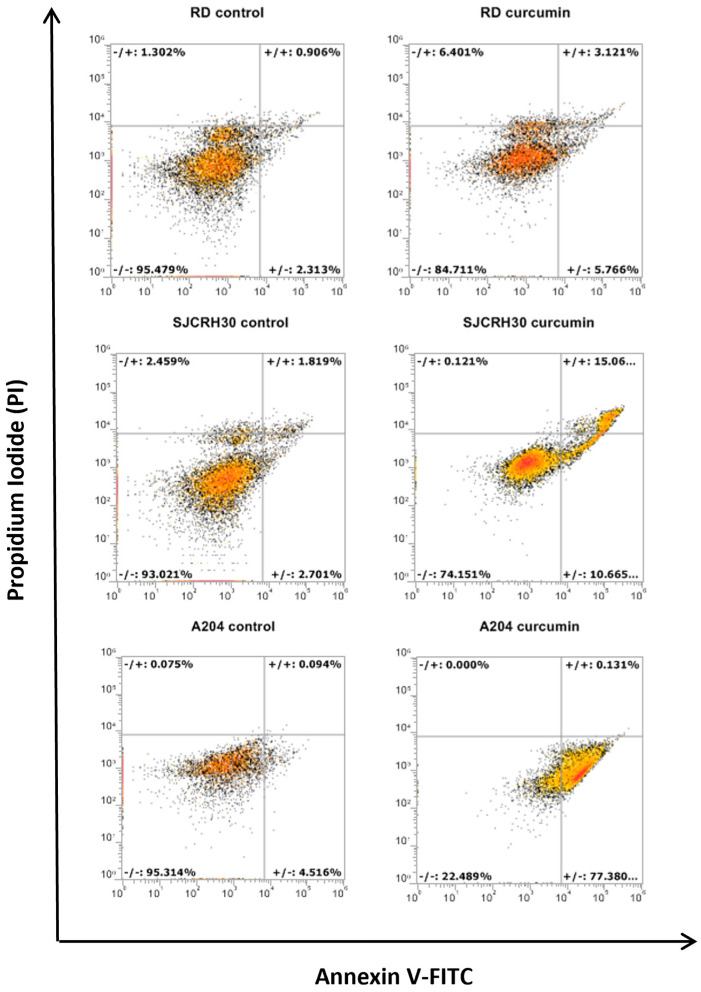
Curcumin treatment induces apoptotic cell death. Rhabdomyosarcoma cell lines (A204, RD, and RH30) were treated for 24 h with 20 μM of curcumin. Cells were stained with Annexin V-FITC/PI and analyzed by flow cytometry. The dot plots are representative of three independent experiments.

Curcumin possesses more than six reactive moieties that may target diverse cell signaling components, leading to cell death. To ascertain by what primary means (apoptosis, necrosis, etc.) curcumin could induce rhabdomyosarcoma cell death, annexin V/PI staining of curcumin treated RD, RH30, and A204 cells was conducted. In each of the cell lines, primary increases in annexin V or annexin V/PI double staining were observed, indicating a prevalently apoptotic means of cell death. Similar to Trypan blue staining, A204 cells showed the most sensitivity to curcumin apoptotic inducing effects, with RD cells demonstrating the most resistance (Figure 2). The fact that apoptotic cell death was also observed in RD and RH30 cells indicated that curcumin induced cell death was not completely dependent on p53, although apoptosis in these cell lines was reduced in comparison to A204 cells.

### 3.3. Curcumin Inhibits Cell Migration and Colony Forming Ability

**Figure 3:**
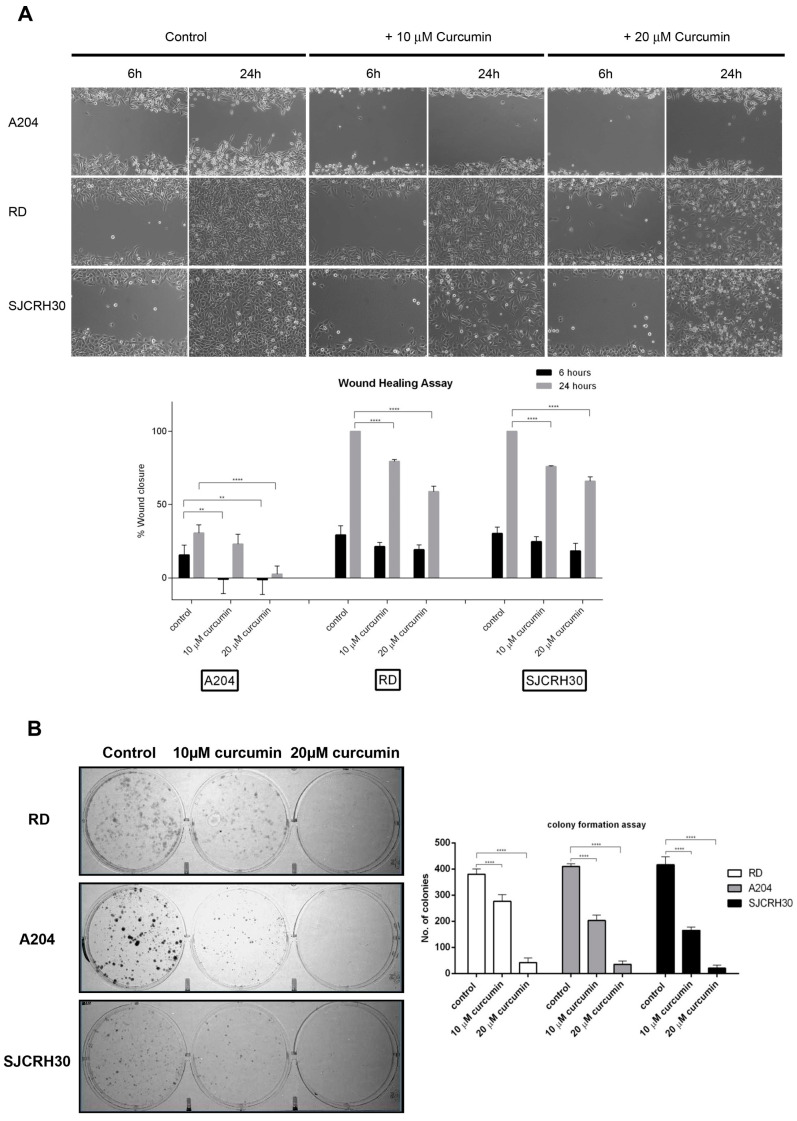
Curcumin influences rhabdomyosarcoma cell migration and colony forming ability. (**A**) Rhabdomyosarcoma cells (A204, RD, RH30) 90% confluence were “wounded” with a 200 μL pipet tip. Three “wounds” were formed on each culture dish in duplicate per experiment. Loose cells were removed with washing and fresh complete medium containing vehicle (DMSO) or curcumin (10 or 20 µM) was added to each cell monolayer. The “wounds” were photographed under light microscopy at 6 and 24 h after the initial “wounding”, and the diameters measured using the AxioVision software. Data were plotted as percent healing of the original “wound” and graphed using GraphPad Prism software. ** *p* < 0.01, **** *p* < 0.0001. (**B**) Rhabdomyosarcoma cells (A204, RD, and RH30) were treated with either vehicle control (DMSO) or curcumin (10 or 20 µM) for 24 h, then plated (500 viable cells/well in 6-well culture plates) and cultured in normal growth media for 10 days. Cells were fixed and stained with crystal violet. Colony number and size were determined using ImageJ software. Results are the mean of three different experiments ±s.d. **** *p* < 0.0001.

A key characteristic of advanced tumors is their ability to migrate to distant sites and establish new metastatic tumor colonies. To assess whether curcumin might affect the metastatic ability of rhabdomyosarcoma, RD, RH30, and A204 cells were examined for their ability to migrate (“wound healing assay”) and form colonies in the presence of curcumin. Curcumin was able to inhibit RMS cell migration in a dose-dependent fashion. Following 24 h of “wound healing”, 20 μM curcumin inhibited cell migration of both RH30 and RD cells by 40% versus the respective control (Figure 3A).

While A204 cells demonstrated poor migratory ability, being unable to close the wound by more than 30% after 24 h in the control, the “wounds” of both RD and RH30 controls were completely closed after 24 h. These cell lines also showed similar migratory inhibition responses to curcumin.

Likewise, curcumin inhibited the colony forming ability of the RMS cell lines in a dose-dependent manner. While 20 μM curcumin resulted in over an 80% reduction in colonies produced by all the tested cell lines, 10 μM curcumin resulted in only 25% reduction in RD colonies, while inducing a 50% reduction in RH30 and A204 colonies (Figure 3B).

### 3.4. Curcumin Treatment Alters Diverse Signal Transduction Pathways Important to Tumor Growth

Previous studies have examined individual pathways altered by curcumin. To our knowledge, this is the first study which uses commercial protein arrays to examine the effects of curcumin on rhabdomyosarcoma. Phospho-kinase array analysis of A204, RH30, and RD cells indicated that curcumin inhibited several previously identified signal transduction intermediates, as well as several novel targets in this pathology.

**Figure 4:**
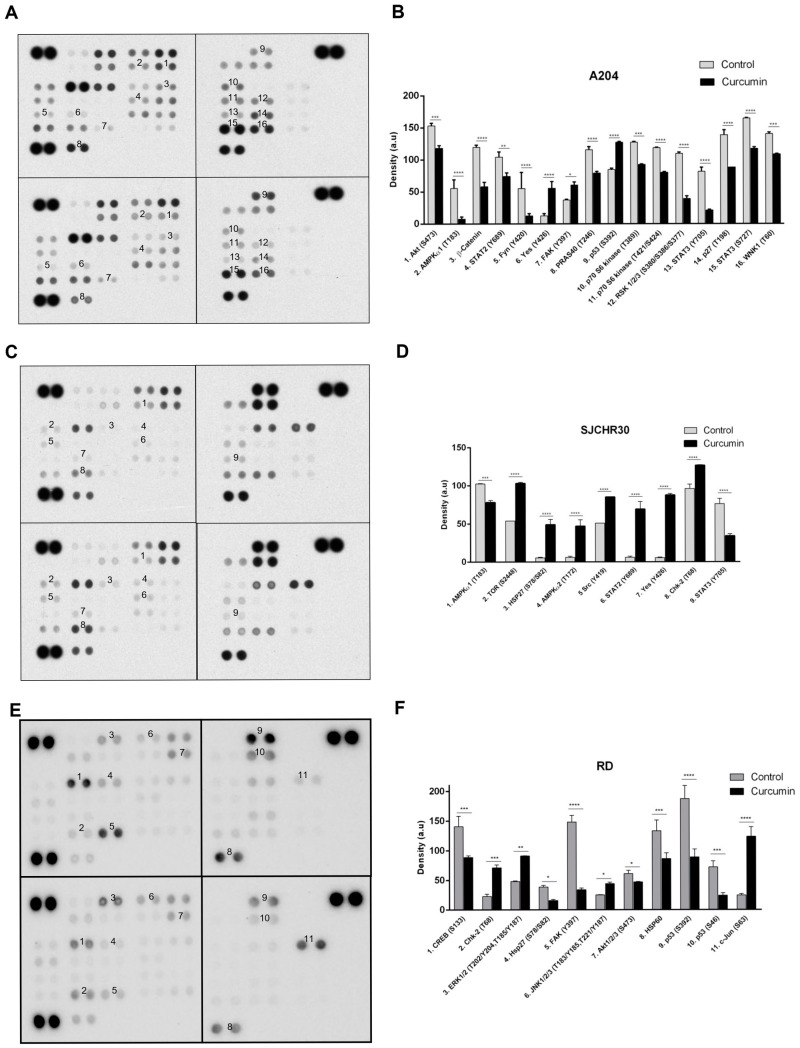
Curcumin influences signaling through AKT/mTOR, STAT, AMPK, and p53 associated pathways. (**A**,**C**,**E**) Whole cell lysates prepared from A204 (**A**,**B**), RH30 (**C**,**D**), and RD (**E**,**F**) cell lines that were treated with DMSO (control) or 20 µM curcumin for 24 h were analyzed using Proteome Profiler Phospho-Kinase arrays. Exemplary array blots are shown. (**B**,**D**,**F**) Percent changes in spot density of Proteome Profiler array proteins/phospho-proteins of interest that were quantified using ImageJ software and normalized to positive controls on the same membrane. * *p* < 0.05, ** *p* < 0.01, *** *p* < 0.001, **** *p* < 0.0001.

Use of the Proteome Profiler Phospho-kinase array indicated that with the exception of phospho- (p)-AMPKα1 (T183), p-AKT (S473), p-YES (Y426), p-CHK2 (T68), and p-STAT3 (Y705) levels, which were altered in two out of three cell lines in response to curcumin treatment for 24 h (control vs. treated), changes in other signaling intermediates were cell line specific (Table 1). Of the common intermediates, levels of p-AMPKα1 (T183) and p-STAT3 (Y705) decreased in both A204 (55.5 ± 12.4 vs. 7.5 ± 3.5; −86% and 81.5 ±6.4 vs. 21 ± 1.4; −74%, respectively), and RH30 cells (102.4 ± 0.6 vs. 78 ± 2.8; −24% and 76 ± 7.1 vs. 34 ± 2.8; −55%, respectively), while decreased levels of p-AKT (S473) were seen in A204 and RD cells (153 ± 4.2 vs. 118 ± 4.2; −23% and 61 ± 5.4 vs. 46.8 ± 0.8; −23%, respectively). Phosphorylation of neither AMPKα1 on T183 nor STAT3 on Y705 were observed in RD cells, while levels of p-AKT (S473) did not significantly change in RH30 cells in response to curcumin. Interestingly, typical effects of AKT inhibition such as decreased β-catenin (119.5 ± 3.5 vs. 58 ± 7.1; −51%), reduced phosphorylation of PRAS40 (115.5 ± 4.9 vs. 79 ± 2.8; −32%; pT246), and reduced phosphorylation of p70S6K (127 ± 1.4 vs. 92.5 ± 0.7; −27% and 118.5 ± 0.7 vs. 80 ± 1.4; −32%; pT389 and pT421/S424, respectively) were only seen in A204 cells and not RD, where their levels either did not change significantly or the level was below the threshold of detection (Figure 4).

Curcumin treatment resulted in increased levels of p-YES (Y426) in both A204 and RH30 cells (12.5 ± 3.5 vs. 55.5 ± 10.6; +344% and 5.5 ± 0.7 vs. 87.5 ± 2.1; +1491%, respectively), while it promoted enhancement of p-CHK2 (T68) levels in RH30 and RD cells (96 ± 5.7 vs. 126.5 ± 0.7; +32% and 22.8 ± 3.7 vs. 71.2 ± 4.7; +212%, respectively). Curcumin treatment also influenced p53 phosphorylation. In A204 cells which harbor wild-type (wt) p53, curcumin induced enhanced phosphorylation of S392 (84.5 ± 2.1 vs. 127 ± 1.4; +50%). In contrast, in the RD cell line, which expresses homozygous mutant p53, curcumin resulted in decreased levels of p-p53 (S46; 72.1 ± 10 vs. 24.2 ± 4.2; −66%) and p-p53 (S392; 187.2 ± 22.1 vs. 88.9 ± 13.2; −53%). RH30 cells, which are heterozygous for mutant p53, maintained elevated levels of both p-p53 (S46) and p-p53 (S392) that were not influenced by curcumin (Figure 4C,D).

Of the tested cell lines A204 demonstrated the most classic response to curcumin’s antitumor effects, with decreases in active (phosphorylated) intermediates of proliferation/survival pathways such as AKT-mTOR-p70S6K, STAT, RSK, and WNK1 and increases in active forms of cell cycle, differentiation, and pro-apoptotic effectors, such as YES and p53. In RH30, other than the previously mentioned decreases in active AMPKα1 and STAT3 levels, curcumin treatment promoted enhanced levels of active signaling intermediates associated with cell survival, growth, autophagy, and inflammation, including AMPKα2, mTOR, STAT2, and SRC along with the aforementioned increases in active levels of cell cycle, differentiation, and pro-apoptotic effectors YES and CHK2. The ERMS cell line RD demonstrated the most interesting profile. While curcumin resulted in decreased levels of active AKT and the downstream transcription factor CREB, it resulted in enhanced MAPK pathway activation (ERK, JNK) and levels of active c-Jun (Figure 4).

## 4. Discussion

The exact originating alterations that lead to rhabdomyosarcoma are still debatable, but previous studies involving fusion-positive and -negative RMS have identified several signaling pathways important for rhabdomyosarcoma progression, invasion, and metastasis. Most RMS are sporadic, but the association of RMS with various germline syndromes of known origin such as Li-Fraumeni syndrome, Noonan syndrome, and neurofibromatosis has implicated the genes p53, NF1, KRAS, NRAS, RAF1, PTPN11, SHOC2, and SOS10 in RMS development [13]. In fusion-positive ARMS, the altered activity of the generated fusion products influences the transcriptional expression and activation of a number of signaling intermediates involved in cell proliferation, survival, and migration. The fusion protein produced in PAX3-FOXO1 ARMS, for example, is known to affect the expression of around 70 mRNAs and 30 miRNAs [13,21]. Transcriptional profiling analysis in ARMS and normal fetal skeletal muscle has identified around 172 differentially expressed genes, while profiling between fusion-positive ARMS and fusion-negative ARMS has identified about 103 differentially expressed genes [13,22]. Regardless of fusion status, the most commonly active signal transduction pathways in RMS are the RAF/MEK/ERK and PI3K/AKT/mTOR pathways, both of which are stimulated downstream of tyrosine kinase receptors (RTKs), such as PDGFR, as well as multiple non-tyrosine kinase receptors, with activation of these pathways occurring in both autocrine and paracrine fashion [13]. Other signaling pathways implicated in past studies involving RMS include IL-4R/JAK/STAT, PDGF and PDGFRα/β, IGFR1/AKT/mTOR, G-protein coupled receptors, NOTCH1, integrin α2β1, Hippo, and WNT/β-catenin [13].

Due to its low biotoxicity profile and reputed health benefits, the spice and herbal supplement curcumin has been extensively studied for its anti-tumorigenic properties, including several in vitro studies examining its efficacy against rhabdomyosarcoma where it was reported to inhibit the proliferation and viability of a pair of tested “RMS” cell lines [17,19]. Unfortunately, due to its poor absorption, rapid degradation, and lack of specificity, the bioavailability of curcumin in its natural state makes it a poor candidate for clinical use [16,18]. The study we report here sought to exploit the non-specific reactivity of curcumin’s various active groups in order to identify potential signal transduction intermediates important to rhabdomyosarcoma growth, survival, and progression that are potential targets for therapeutic intervention and drug-design. Similar to previous reports, the data demonstrate that curcumin is effective at inhibiting RMS cell proliferation and viability. While previous IC50 values for curcumin in RMS cell lines have been reported to be around 2.5 µM, our data indicated IC50 values between 10 and 25 µM, depending on the tested cell line. This discrepancy is likely due to the fact that the IC50 values reported here relate to 50% cell viability, while in earlier studies the reported IC50 values referred to cell growth [19]. Likewise, earlier studies reported effects obtained after 6 days, while those reported in this study were obtained after 3 days. Interestingly, in our study, curcumin was found to preferentially induce a G2/M block in the cell cycle and apoptotic cell death. Previous studies using the cell lines RH1 and RH30 (also used in this study) reported that curcumin induced a G1 phase cell cycle arrest [19]. While we are unsure of the reason for this discrepancy between previous findings and ours, part of the reason for these differences may lie in cell line selection. Originally thought to be rhabdoid in origin, the RH1 cell line used in the earlier study is now known to be an Ewing sarcoma [20,23]. Additionally, the G2/M arrest we report in this study was observed in each of the three RMS cell lines evaluated (RD, A204, and RH30). Moreover, curcumin treatment was not only found to inhibit cell migration, as was previously reported [19], but it also reduced the colony forming ability of the RMS cell lines. Whereas prior studies using curcumin in rhabdosarcoma centered around a critical role for mTOR-mediated signaling in the growth and survival of rhabdomyosarcoma, data obtained from proteome profiling, using a phospho-kinase array, indicated that in ERMS (A204 and RD) in addition to inhibition of mTOR—most specifically through inhibiting the phosphorylation of AKT on S473 (A204 and RD) and PRAS40 on S246 (A204)—curcumin’s effects also extended to changes in the phosphorylation/activation of AKT/mTOR downstream intermediates p70S6K (A204), WNK1 (A204), and CREB (RD), as well as β-catenin expression (A204). In contrast to A204, RD cells demonstrated an upregulation of ERK and JNK activity in addition to diminished active AKT. Of significant interest was the role of p53. In A204 cells which express wild-type p53, curcumin resulted in enhanced levels p53 phosphorylated on S392, a site which favors both enhanced p53 transcriptional activity as well as protein stability [24,25,26,27,28], indicative of a role for p53 in curcumin-induced cell death in this cell line. In contrast, curcumin treatment of RD cells, which contain a homozygous mutation in p53 and express a mutant p53 protein, promoted the opposite. Levels of p53 phosphorylated on S46 and S392 were found to decrease in response to curcumin. In conjunction with decreased AKT activity that would be expected to result in enhanced MDM2 activity, these findings would suggest favorable conditions for the instability and degradation of mutant p53 expressed in RD. Thus, in RD cells, mutant p53 is likely actively participating in the tumorigenic process by acting as an oncogene [29,30]. This would suggest that in rhabdomyosarcoma tumors expressing oncogenic mutant p53 protein, targeting the degradation of p53 may be a relevant approach to treating these cancers. While the ARMS cell line RH30 harbors a heterozygous p53 mutation, p53 evidently does not play a significant role in curcumin induced cell death in this cell line as these cells maintain a continuously elevated amount of p53 phosphorylated on S46 and S392 that was not influenced by curcumin treatment. Instead, the profile of RH30 indicated a shift toward the integrated stress response (ISR) and autophagy with the upregulation of active STAT2, mTOR, and AMPKα2, while curcumin resulted in the reduction of active AMPKα1 and STAT3. The involvement of AMPK and JAK/STAT pathway members in A204 and RH30 is rather intriguing. AMPK is composed of α, β, and γ subunits; it is the α-subunit that contains the catalytic domain of the kinase [31,32,33]. Two α-subunits exist, α1 and α2. Both AMPKα1 and AMPKα2 respond to energy stress and serve a protective role for the cell against temporary deficiencies of energy in the form of ATP. Activation favors the shutdown of energy expending cellular processes while at the same time promoting pathways favoring energy production such as increased glucose receptor synthesis, synthesis of lipases, and autophagy [34,35]. However, whereas AMPKα1 is ubiquitously expressed, AMPK2α is more highly expressed in muscle, kidney/bladder and brain, and acts as the main effector during basal and induced AMPK activity [36]. Additionally, AMPKα2 is more closely associated with the regulation of mitophagy, a specialized form of autophagy, in cardiac muscle [37]. Reduced activity of both kinases would result in unchecked metabolic stress for the cell (A204), while the suppression of AMPKα1 activity in the presence of enhanced AMPKα2 activity (as observed RH30) would be expected to favor autophagy. Autophagy may be further modulated based on the status of the JAK/STAT pathway member STAT3. Transcriptionally-active STAT3 is phosphorylated on Y705 in response to a number of interleukins and growth factors, and upon phosphorylation translocates to the nucleus where it stimulates genes involved in inflammation, cellular homeostasis, and cell cycle progression [38,39]. Reduced phosphorylation of STAT3 on Y705, as observed in both A204 and RH30, favors the retention of STAT3 in the cytoplasm, not only inhibiting its transcriptional activity but also inhibiting induction of macroautophagy through the suppression of the integrated stress response/inflammatory kinase PKR [40]. Additionally, levels of transcriptionally active STAT2 were differentially affected by curcumin treatment in A204 and RH30. Whereas, similarly to STAT3, curcumin treatment resulted in reduced phosphorylation of STAT2 on Y689 in A204 cells, it resulted in an increase in transcriptionally-active STAT2 in RH30 cells. Unlike STAT3, STAT2 is more strongly associated with interferon signaling and the establishment of an antiviral state. Among the interferon-inducible genes that are induced by STAT2 is PKR [38,41].

The results obtained with curcumin in this study suggest several novel approaches to achieve a more effective therapy for rhabdomyosarcoma by targeting AKT-mTOR, AMPK, STAT, and mutant p53. Previous studies have shown promise by targeting the PI3K/AKT/mTOR pathway in rhabdomyosarcoma [10,42,43,44,45,46,47,48]. A number of type I PI3K inhibitors have demonstrated significant success in recent years and are currently in clinical trials. We recently reported that combination therapy with two FDA and EMA approved type I PI3K inhibitors targeting p110α and p110δ showed enhanced therapeutic potential in RMS at reduced concentrations, opening the door to possible phase trials to evaluate the clinical effectiveness [49]. Additionally, the presence of STAT family members, especially STAT3, as potential targets is interesting as STAT3 signaling has been found to be constitutively activated in RMS, and the small molecule inhibitors LLL12 and FLLL32 were reported to inhibit STAT3 activity, and inhibit the proliferation and viability of rhabdomyosarcoma cell lines more efficiently than a number of previous JAK/STAT pathway inhibitors, as well as curcumin [50].

Data from the study presented here suggest that in addition to targeting the PI3K/AKT/mTOR pathway in ERMS, additional combinational targeting of AMPK and STAT signaling with specific small molecule inhibitors, or more efficacious curcumin derivatives in development [51], may offer significant promise in treating this aggressive pathology. Moreover, the presence of oncogenic p53 should be assessed as these mutants may offer a secondary therapeutic target which can enhance the efficiency of primary therapeutics. In fusion-positive ARMS, forcing autophagic processes may serve as an adjunct therapy to conventional means such as radiation. Past findings demonstrated that interferon treatment was able to enhance the effects of radiation in ARMS tumors in mice [52]. Interferon treatment would be expected to have induced STAT2 transcriptional activation, similar to what was observed in the RH30 cell line following curcumin treatment. Thus, STAT2 activation together with AMPK activation or mTOR inhibition might prove effective for these tumors.

While the reported study is limited to in vitro analysis, due to the poor bioavailability and biostability of curcumin, it clearly demonstrates an anti-tumorigenic role of curcumin in rhabdomyosaroma. Additionally, beyond characterizing a single signaling pathway or intermediate in the induced response, the data present a comprehensive signaling profile in response to curcumin treatment in three cell lines that differ based on the type of rhabdomyosarcoma (ARMS vs. ERMS), p53 status, and associated oncogenic mutations. Unfortunately, while the study design did not allow for the direct identification of curcumin’s specific targets, it does present a unique tumor specific profile of major signaling pathways affected by curcumin treatment, indicating both cell line associated tumor-specific effects, as well as common effects, that can be further exploited for therapeutic drug design.

## 5. Conclusions

The results from this study not only verify the previously reported anti-tumorigenic properties of curcumin in rhabdomyosarcoma, but they also open the door to the testing of small molecule inhibitors to AMPK, PI3K/AKT/mTOR, STAT, and p53, individually or in combination depending on the characteristics of the rhabdomyosaroma in question (ARMS or ERMS, wt p53, or mut p53), in vitro and in vivo. In addition, the data obtained aid in the establishment of a curcumin signaling profile in rhabdomyosarcoma which can be used for the potential validation of more bio-active and -stable derivatives of curcumin in development. Future studies will aim to assess both these aspects in cell culture and mouse models.

## Figures and Tables

**Table 1:** Changes in signal transduction mediators observed in RMS cell lines following curcumin treatment.

Protein (Modification)	A204	RH30	RD	Result/Process
**AMPKα1 (T183)**	**↓**	**↓**		(**Activation, intracellular localization**) The catalytic subunit of the cellular energy sensing protein kinase AMP-activated protein kinase (AMPK). Inhibits energy consuming cellular processes (transcription, translation, ribosome biogenesis, etc.) under conditions of low ATP. Enhances glucose uptake and regulates insulin signaling and glycolysis. Enhances transcriptional activity of transcription factors involved in regulating energy metabolism. Directly inhibits the mTORC1 complex under low nutrient conditions. Promotes autophagy. Regulates cell polarity. Regulates lipid biosynthesis. Phosphorylates and stabilizes β-catenin. Phosphorylation at this site leads to activation and can occur through AMP/ATP-dependent means as well as -independent.
**AMPKα2 (T172)**		**↑**		(**Activation, intracellular localization, protein degradation**) The catalytic subunit of the cellular energy sensing protein kinase AMP-activated protein kinase (AMPK). Inhibits energy consuming cellular processes (transcription, translation, ribosome biogenesis, etc.) under conditions of low ATP. Enhances glucose uptake and regulates insulin signaling and glycolysis. Enhances transcriptional activity of transcription factors involved in regulating energy metabolism. Directly inhibits the mTORC1 complex under low nutrient conditions. Promotes autophagy. Regulates cell polarity. Regulates lipid biosynthesis. Phosphorylates and stabilizes β-catenin. Phosphorylation at this site leads to activation and can occur through AMP/ATP-dependent means as well as independent.
**Akt (S473)**	**↓**		**↓**	(**Activation, intracellular localization**) Ser/Thr kinase involved in cell proliferation, survival, and metabolism. Regulates glucose uptake. Is activated by insulin and insulin-like growth factor (IGF). Activation and subsequent phosphorylation and inhibition of the downstream kinase GSK3 promotes the storage of glucose in the form of glycogen. AKT kinases are activated in response to a wide range of growth factors, cytokines, and stress leading to enhancement of mTOR-dependent signaling, activation of NF-κB-dependent gene transcription, and stimulation of CREB1-dependent transcription. Regulates Forkhead box protein transcription factors. Promotes β-catenin stabilization through the inhibition of GSK3B. Promotes the synthesis of anti-apoptotic proteins and results in the sequestering and degradation of pro-apoptotic proteins. Phosphorylation on this site is required for full activity.
**β-Catenin**	**↓**			Transcriptional regulator of the WNT pathway. Under conditions favoring GSK3B activation, β-catenin is phosphorylated, ubiquitinated, and degraded. In the presence of WNT or the inhibition of GSK3B kinase activity, β-catenin is not degraded but accumulates in the nucleus where it serves as a coactivator for transcription. Regulates cell adhesion and anchorage-independent growth, promotes neurogenesis, regulates insulin internalization.
**Chk-2 (T68)**		**↑**	**↑**	(**Activation, intracellular localization**) Ser/Thr kinase required for cell cycle checkpoint arrest primarily at the G2/M transition point. Has a role in DNA repair and apoptotic cell death induced by DNA double-strand breaks. Phosphorylation at this site enhances homodimerization and subsequently full kinase activation.
**CREB (S133; i.e., S119)**			**↓**	(**Transcriptional activation**) Transcription factor which binds to the cAMP response element present in many promoters of cellular and viral genes. Involved in diverse cellular processes.
**ERK1/2 (T202/Y204, T185/Y187)**			**↑**	(**Activation, intracellular localization**) Ser/Thr kinases central to the MAP kinase family. Have diverse roles in proliferation, transcription, translation, survival, cell cycle/cell division. Directly phosphorylates and regulates several transcription factors and translation regulatory proteins.
**FAK (Y397)**	**↑**		**↓**	(**Activation, intracellular localization, protein degradation**) Non-receptor protein tyrosine kinase involved in the regulation of cell adhesion, cell spreading, cell migration, cell proliferation, cell cycle progression, actin cytoskeletal rearrangements, and apoptosis. Required for normal embryonic development (angiogenesis, heart development, and nervous system development) and osteogenesis. Acts downstream of membrane receptors (cytokine, chemokine, growth factor, integrin, G-protein coupled, and immune) where it becomes tyrosine phosphorylated and associates in complex with Src tyrosine kinase family members. Additional tyrosine phosphorylation of PYK2 transforms it into a scaffolding protein capable of stimulating PI3K/AKT/mTOR, RAS/RAF/MEK/ERK and SAPK/JNK1 pathways as well as the translocation of MDM2 to the nucleus where it leads to p53 degradation. Phosphorylation or autophosphorylation at Y397 creates a docking site for SRC kinase family members that are then responsible for subsequent phosphorylation of Y576 and Y577 to induce full FAK activity.
**Fyn (Y420)**	**↓**			(**Intermolecular association**) Non-receptor protein tyrosine kinase. Has a role in regulating cell growth and survival, immune response, cell motility, remodeling of the cytoskeleton, and integrin signaling. Following phosphorylation at the C-terminus associates with focal adhesion kinase FAK1 allowing for FAK1 phosphorylation and activation. Phosphorylates β- and Δ-catenins to regulate cellular adhesions. Promotes T-cell differentiation following binding of the T-cell receptor (TCR) through a mechanism involving focal adhesion kinase PYK2. Responsible for CD28 stimulation induced VAV1 activation. Activation of FYN is inhibited by phosphorylation at Y531 and activated by its dephosphorylation. Autophosphorylation of Y420 is required for full activation.
**HSP27 (S78/S82)**		**↑**	**↓**	(**Activation, intracellular localization, protein degradation**) Heat shock protein which functions as a molecular chaperone. Has a role in stress resistance by assisting proteins to maintain the correct folded state. Stress-induced phosphorylation at S78 and S82 impairs chaperone activity.
**HSP60**			**↓**	A chaperonin involved in mitochondrial protein import. It also serves for correct folding of mitochondrial matrix proteins following stress.
**JNK (T183/Y185, T221/Y223)**			**↑**	(**Activation**) Involved in cell proliferation, transformation, migration, differentiation, and death. Activated in response to proinflammatory cytokines or cellular stress. Through interaction and phosphorylation of AP-1 transcription factor components, leads to activation of AP-1-dependent transcription. Inhibits replication initiation (JNK1). Inhibits rRNA synthesis upon ribotoxic stress by inactivating RNA pol I (JNK2). Promotes stress induced cell apoptosis by phosphorylating p53 and YAP1. Necessary for Th1 polarization of T-helper cells. Promotes the degradation of β-catenin (JNK2). Can promote the activation of autophagic pathways (JNK1). JNK3 is specific to cells of the nervous system.
**c-Jun (S63)**			**↑**	(**Acetylation, transcriptional activity induced, protein stabilized**) Transcription factor that is a component of the AP-1 transcription factor family. Activation stimulates multiple genes regulating diverse cellular processes.
**p27 (T198)**	**↓**			(**Altered protein stability, altered intracellular localization**) Regulates cell cycle progression. Inhibits cyclin A- and cyclin E-CDK2 complexes but promotes cyclin D-CDK4 complexes depending on its phosphorylation (not only at this site).
**p53 (S392)**	**↑**		**↓**	(**Transcriptional activation, intracellular localization, altered protein stability**) Transcription factor involved in DNA repair, apoptosis, and cell cycle regulation. Regulates the circadian clock. Regulates early ribogenesis. Activated in response to stress. Phosphorylation of S392 has been linked to p53 protein stability and transcriptional activity.
**p53 (S46)**			**↓**	(**Transcriptional activation, intracellular localization, altered protein stability**) Transcription factor involved in DNA repair, apoptosis, and cell cycle regulation. Regulates the circadian clock. Regulates early ribogenesis. Activated in response to stress.
**p70 (T389)**	**↓**			(**Activation, intracellular localization**) Ser/Thr kinase activated downstream of mTOR in response to growth promoting stimuli. Phosphorylates various downstream targets to regulate protein synthesis at the level of initiation and elongation. Has a feedback regulatory role on mTORC1/2 signaling. Has a role in promoting TNFα-induced insulin resistance.
**p70 (T421/S424; i.e., T444/S447)**	**↓**			(**Activation**) Ser/Thr kinase activated downstream of mTOR in response to growth promoting stimuli. Phosphorylates various downstream targets to regulate protein synthesis at the level of initiation and elongation. Has a feedback regulatory role on mTORC1/2 signaling. Has a role in promoting TNFα-induced insulin resistance.
**PRAS40 (T246)**	**↓**			(**Activity inhibited**) A negative regulatory subunit of the mTORC1 complex. Negative regulatory activity is relieved by phosphorylation on T246.
**RSK1/2/3 (S380/S386/S377)**	**↓**			(**Activation, protein degradation, ubiquitination**) Ser/Thr kinases downstream of ERK1/ERK2 responsible for regulation of proliferation, survival, and differentiation by modifying mTOR-dependent signaling. Directly influences factors regulating transcription and translation in response to mitogenic- or stress-mediated stimulation. Inhibits GSK3β activity by phosphorylating Ser9 of GSK3B. Promotes assembly of the translational preinitiation complex. Enhances CAP-dependent translation through phosphorylation of EIF4B. May suppress mTOR activity by phosphorylating TSC2. Involved in cell cycle regulation. Regulates osteoblast differentiation. Sites S380 (RSK1), S386 (RSK2), and S377 (RSK3) are activation promoting autophosphorylation sites.
**Src (Y419)**		**↑**		A proto-oncogenic non-receptor tyrosine kinase which has a role in numerous signaling events in the cell. Is a central component of the Src receptor signaling complex.
**STAT2 (Y689)**	**↓**	**↑**		(**Intracellular localization**) Signal transducer and activator of transcription. Mediates signaling by type I interferons (IFNs). Important for inducing the antiviral state. It also acts as a negative feedback regulator of IFNAR2. Acts as a regulator of mitochondrial fission. Phosphorylation of Y689 favors nuclear localization.
**STAT3 (Y705)**	**↓**	**↓**		(**Activation, intracellular localization, methylation, protein degradation**) Signal transducer and activator of transcription. Mediates cellular responses to diverse interleukin (ILs) and growth factor receptor stimulation. Helps recruit coactivators of transcription to target genes. Involved in the T-cell inflammatory response. Cytoplasmic STAT3 can inhibit activation of the integrated stress response kinase PKR. Regulates β-cell insulin secretion. Phosphorylation at Y705 induces nuclear localization and transcriptional activation.
**STAT3 (S727)**	**↓**			(**Activity altered, intracellular localization**) Signal transducer and activator of transcription. Mediates cellular responses to diverse interleukin (ILs) and growth factor receptor stimulation. Helps recruit coactivators of transcription to target genes. Involved in the T-cell inflammatory response. Cytoplasmic STAT3 can inhibit activation of the integrated stress response kinase PKR. Regulates β-cell insulin secretion. Phosphorylation at S727 is required for maximal transcriptional activation by enhancing DNA binding.
**TOR (S2448)**		**↑**		(**Activation; intracellular localization**) The central catalytic subunit of the mTORC1 and mTORC2 kinase complex, which act as major regulators of cellular growth, survival, and metabolism in response to nutrients, stress, energy, and growth factor stimulation. Is a downstream target of the PI3K-AKT pathway. Promotes protein synthesis through the phosphorylation of the eukaryotic initiation factor (eIF)-4E binding protein (4EBP) and by promoting the modification of ribosomal S6 protein. Stimulates ribosome biogenesis by enhancing RNA pol III activity. Regulates autophagy through the phosphorylation of ULK1 and DAP.
**WNK1 (T60)**	**↓**			(**Activation**) Ser/Thr kinase, regulates Na^++^/K^++^-chloride coupled receptors. Has a role in cytoskeletal reorganization.
**Yes (Y426)**	**↑**	**↑**		(**Activation**) Non-receptor tyrosine kinase (non-RTK) stimulated downstream of various RTKs. Involved in cell growth, survival, apoptosis, cell adhesion, cytoskeletal remodeling, and differentiation. Regulates G1 and G2/M phases of the cell cycle. Required for AKT-mediated cell migration. Phosphorylation at Y426 blocks inhibitory phosphorylation.

Information presented was obtained from the PhosphositePlus database (http://www.phosphosite.org, accessed 11 November 2022) and the UniProtKB/SwissProt database (http://www.uniprot.org, accessed 14 November 2022). Arrows in each row indicate whether the expression or phosphorylation of each indicated protein or modification was either upregulated (**↑**), downregulated (**↓**) or unchanged (blank).

## Data Availability

The data presented in this study are available upon request.
